# Weeds as Potential Inoculum Reservoir for *Colletotrichum nymphaeae* Causing Strawberry Anthracnose in Iran and Rep-PCR Fingerprinting as Useful Marker to Differentiate *C. acutatum* Complex on Strawberry

**DOI:** 10.3389/fmicb.2019.00129

**Published:** 2019-02-12

**Authors:** Kaivan Karimi, Mahdi Arzanlou, Ilaria Pertot

**Affiliations:** ^1^Department of Plant Protection, Faculty of Agriculture, University of Tabriz, Tabriz, Iran; ^2^Department of Sustainable Agro-Ecosystems and Bioresources, Research and Innovation Centre, Fondazione Edmund Mach, San Michele all'Adige, Italy; ^3^Center Agriculture Food Environment (C3A), University of Trento, San Michele all'Adige, Italy

**Keywords:** *Fragaria* × *ananassa*, anthracnose, asymptomatic plants, inoculum reservoir, molecular identification, kurdistan province

## Abstract

Strawberry anthracnose caused by *Colletotrichum* spp. is considered one of the most serious and destructive disease of strawberry worldwide. Weeds, as possible hosts of the pathogen, could have a role as potential inoculum reservoir. To prove this hypothesis, symptomless weeds were collected in strawberry fields showing anthracnose symptoms in Iran. Ten isolates with *Colletotrichum*-like colonies were recovered from symptomless *Amaranthus viridis* L., *Convolvulus arvensis* L., *Fumaria officinalis* L., *Lactuca serriola* L., and *Sonchus oleraceus* L. plants. The isolates were identified as *C. nymphaeae*, based on a combination of morphological and sequence data of TUB and GADPH genes. This identification was further validated using Rep-PCR fingerprinting analysis, which produces species-specific DNA fingerprints and unveils inter and intra variation of the species examined in this study. Moreover, rep-PCR marker was used to reveal accurate taxonomic position of *Colletorichum* spp. causing strawberry anthracnose belonging to the *C. acutatum* complex, including *C. acutatum sensu stricto, C. fiorinae, C. godetiae, C. nymphaeae, C. salicis*, and *C. simmondsii*. The *C. nymphaeae* isolates originating from symptomless weeds confirmed their pathogenicity on detached strawberry, proving that weeds in strawberry field may have a role as reservoir of inoculum. However, further studies are necessary to quantify their actual contribution to anthracnose epidemics in strawberry fields.

## Introduction

*Colletotrichum* spp. are among the most widespread and destructive phytopathogenic fungi worldwide. The inclusion of these species among the top 10 fungal pathogens indicates their economic importance and also justifies the interest of scientists (Dean et al., 2012). Most *Colletotrichum* spp., *viz*. *C. acutatum, C. gloeosporioides, C. dematium, C. destructivum, C. gigasporum, C. boninense* and *C. orbiculare*, are known as complex species comprising different cryptic species (Damm et al., 2009, 2012a,b, 2013, 2014; Weir et al., 2012; Liu et al., 2014). Nearly all crops worldwide are susceptible to one or more species of *Colletotrichum* (Dean et al., 2012). Strawberry is an important and valuable crop and it is frequently infected by *Colletotrichum* spp. (Freeman and Katan, 1997; Karimi et al., 2017). Anthracnose, the main disease symptom caused by these fungal pathogens, was initially used to name the destructive disease of strawberry caused by *Colletotrichum fragariae* Brooks (Howard et al., 1992). Three species *C. acutatum, C. gloeosporioides* and *C. fragariae*, are commonly indicated as the causal agents of anthracnose on strawberry (Howard et al., 1992; Freeman and Katan, 1997). However, the advent of new molecular approaches, such as multi-gene approach in fungal taxonomy, combined with traditional diagnostic methods (polyphasic approach), revealed that *C. acutatum* and *C. gloeosporioides* are complex species, comprising several species causing anthracnose symptoms on strawberry (Damm et al., 2012a; Weir et al., 2012; Karimi et al., 2017). Nowadays, multi-gene technique is mainly used as the most confident approach to reveal cryptic species among complex species of fungi, particularly *Colletotrichum* spp. (Damm et al., 2009, 2012a, 2014). However, this method is expensive and not accessible for many mycologists all over the world. Therefore, alternative PCR-based genomic fingerprinting, such as rep-PCR, can be very useful to resolve cryptic species. Rep-PCR fingerprinting was originally developed to amplify sequences located between repetitive elements in prokaryotic genomes. However, various studies proved that this molecular marker is capable to provide the fingerprinting of eukaryotic genomes, including fungi (Mehta et al., 2002; Godoy et al., 2004; Alves et al., 2007).

*Colletotrichum nymphaeae*, one of the members of *C. acutatum* complex species, seems to be the most common and devastating *Colletotrichum* sp. on strawberry worldwide, including Iran (Damm et al., 2012a; Baroncelli et al., 2015; Karimi et al., 2017). Besides strawberry, this pathogen can cause anthracnose on other important crops, namely apple, pepper, grapevine and celery (Velho et al., 2014; Yamagishi et al., 2015; Liu et al., 2016; Nasehi et al., 2016). *Colletotrichum nymphaeae* has become a serious problem, threatening strawberry industry in Iran, since its first outbreak in 2013 (Karimi et al., 2017). Different conventional methods, including chemical fungicides, crop sanitation, cultural methods, and resistant cultivars, are used to control the disease (Smith, 2013). It was originally assumed that the primary infection in strawberry fields starts from infected plants growing near the strawberry field (Smith, 2013), since most of the *Colletotrichum* spp. such as *C. nymphaeae* may infect many other hosts than strawberry, including common weeds. Therefore, if this hypothesis is true, the identification and eradication of alternative hosts harboring the pathogen in order to remove pathogen inoculum could become a crucial agronomic practice to prevent infections of strawberry plants. The fact that many alternative hosts, including weeds, may not show any typical symptoms of anthracnose disease due to endophytic nature of *Colletotrichum* spp., could let growers to underestimate the risk. Limited knowledge is available on the potential role of weeds in the epidemiology of *Colletotrichum acutatum sensu lato* in strawberry anthracnose (Berrie and Burgess, 2003). In particular, (Berrie and Burgess, 2003) noticed that the pathogen can overwinter on weeds and survive on infected crop debris in absence of strawberry.

In our study, we isolated a *Colletotrichum* sp. from symptomless common weeds across the anthracnose-infected strawberry fields in Iran (Kurdistan province) and we compared them with type strains of *C. nymphaeae, C. acutatum sensu stricto, C. godetiae, C. fioriniae, C. salicis* and *C. simmondsii*, belonging to the *C. acutatum seneu lato*, in terms of morphological, molecular and pathogenicity.

## Materials and Methods

### Sampling and Isolation

The strawberry fields across Sanandaj and Kamyaran counties (Supplementary Figure 1) (two main areas of strawberry production in Kurdistan province, Iran), were inspected from May to June 2017. In this study, anthracnose-infected fields were identified and weeds were collected inside and around those fields with focusing mostly on broad-leaved weeds, because it has been previously determined that *C. acutatum sensu lato* can be mainly found on those plants (Berrie and Burgess, 2003). Symptomless common weeds across anthracnose-infected strawberry fields were collected and transferred to the lab inside facial bags. In the lab, different plant parts of each sample (leaf, stem, and root) were superficially disinfected by immersion in 90 % ethanol for 1 min, 1 % solution of sodium hypochlorite for 4 min and rinsed three times in sterile distilled water. All plant parts were left to dry on sterile Whatman paper under laminar flow hood. The plant parts were then cultured on potato dextrose agar (PDA; Sigma Aldrich, UK) and monitored for fungal growth on a daily base. Single spore cultures were established on water agar as described by Arzanlou et al. (2015) from colonies growing on PDA. Purified isolates were long term stored on home-made potato carrot agar (20 gr potatoes, 20 gr carrots, 20 gr technical agar per liter) at 4°C for further investigations. Fungal type strains of *C. nymphaeae* (CBS 515.78), *C. acutatum sensu stricto* (CBS 112996), *C. godetiae* (CBS 133.44), *C. fioriniae* (CBS 128517), *C. salicis* (CBS 607.94), and *C. simmondsii* (CBS 122122) were purchased from Westerdijk Fungal Biodiversity Institute, The Netherlands.

### Morphological Description

Macro morphological features of fungal isolates including colony characterization and growth rate were studied on PDA, malt extracts agar (MEA; Sigma Aldrich, UK) and oat meal agar (OA; Sigma Aldrich, UK) as described by Karimi et al. (2017). Color charts of Rayner (1970) was used to rate the color of colonies.

### DNA Extraction, Amplification, And Sequencing

Total genomic DNA of fungal isolates was extracted using a Fast DNA spin kit for soil (MP Biomedical, USA), according to the manufacturer's instructions. DNA quantification was determined using NanoDrop 8000 spectrophotometer (Thermo Scientific, USA) and proportional dilutions were made. The genomic regions of *beta-tubulin* (TUB) and *glyceraldehyde 3-phosphate dehydrogenase* (GAPDH) were amplified with PCR, using the primer sets T1/Bt-2b (Glass and Donaldson, 1995; O'Donnell and Cigelnik, 1997) and GDF1/GDR1 (Guerber et al., 2003), respectively. The reaction mixture of the PCR was prepared in a total volume of 25 μl by mixing 1 μl (50 ng/ μl) of extracted DNA, 1 μl (0.2 μM) of forward and reverse primers, 9.5 μl of nuclease free sterile water and 12.5 μl of DreamTaq Green PCR Master Mix (Fermentase). PCR for TUB and GAPDH was carried out in a Biometra T Professional thermal cycler, as described by Karimi et al. (2016): an initial denaturation at 94°C for 5 min, followed by 40 cycles of 30 s at 95°C, 30 s at 52°C and 30 s at 72°C and final extension at 72°C for 7 min. Amplified products were visualized on 1% agarose gel stained with ethidium bromide. The products were then purified with Exo-Sap enzyme (Euroclone S.p.a., Italy), according to the manufacturer's instructions and sequenced using a BigDye Terminator v3.1 Cycle Sequencing Kit (Applied Biosystems, USA).

### Phylogenetic Analysis

Consensus sequences were established from raw trace files of forward and reverse primers using Staden package program ver. 2.0.0b9 (Staden, 1996). The assembled sequences were subjected to BLAST searches of the GenBank database hosted by NCBI as queries. Two alignments were made for each of the two markers used in this study, by comparing downloaded sequences with the highest similarity and sequences of type species obtained from relevant literature for *C. acutatum* complex (Damm et al., 2012a). Sequences were then aligned with the Muscle software (Edgar, 2004) implemented in Mega6 (Tamura et al., 2013) and manually checked when necessary. Multi-gene analyses were performed using concatenated datasets of TUB and GAPDH made by Mesquite software (Maddison and Maddison, 2018).

For each alignment, the best evolutionary model was calculated using the software MrModelTest v. 2.3 (Nylander, 2004). All analyses were performed using MrBayes v. 3.2.1 (Ronquist and Huelsenbeck, 2003), with heating parameter set at 0.15, and four MCMC running up to 1000000 generations and sampling trees every 1,000 generations. Satisfactory convergence was assessed using the standard deviation of split frequency. The first 25% of saved trees were discarded as the burn-in, and consensus trees and their posterior probabilities (PP) were determined from the remaining trees. The generated phylogenetic trees were inspected and printed using FigTree v. 1.3.1 (Rambaut, 2009). Two strains of *C. orchidophilum* (CBS 128556 and CBS 632.80) were used as out-group taxa during phylogenetic analysis (Damm et al., 2012a). Sequences derived from this study were deposited at NCBI's GenBank nucleotide database (http://www.ncbi.nlm.nih.gov; Table 1).

**Table 1:** *Colletotrichum* spp. associated with strawberry anthracnose used in multi-gene analysis in this study, host/substrate, and country of isolation and GenBank accessions.

**Species**	**Accession no.^**1**^**	**Host/substrate**	**Country**	**GenBank no**.	
				**GAPDH**	**TUB2**
*C. acutatum*	CBS 112996^*^	*Carica papaya*	Australia	JQ948677	JQ005860
*C. fioriniae*	CBS 128517^*^	*Fiorinia externa* (elongate hemlock scale, insect)	USA	JQ948622	JQ949943
	CBS 127611	*Fragaria × ananassa*	USA	JQ948658	JQ949979
	CBS 128529	*Fragaria × ananassa*, root	New Zealand	JQ948661	JQ949982
	IMI 345575	*Fragaria × ananassa*, lesion	New Zealand	JQ948662	JQ949983
	IMI 345578	*Fragaria × ananassa*	New Zealand	JQ948664	JQ949985
	IMI 345583	*Fragaria × ananassa*, lesion	New Zealand	JQ948663	JQ949984
*C. godetiae*	CBS 133.44^*^	*Clarkia hybrida*, cv. Kelvon Glory, seed	Denmark	JQ948733	JQ950053
	CBS 125972	*Fragaria × ananassa*	Netherlands	JQ948747	JQ950067
	CBS 126503	*Fragaria × ananassa*	UK	JQ948751	JQ950071
	CBS 126516	*Fragaria × ananassa*, fruit rot	Netherlands	JQ948749	JQ950069
*C. nymphaeae*	CBS 515.78^*^	*Nymphaea alba*, leaf spot	Netherlands	JQ948527	JQ949848
	CBS 122110	*Fragaria* × *ananassa* cv. Redchief fruit rot	Bulgaria	JQ948565	JQ949886
	CBS 125958	*Fragaria* × *ananassa*, seed	Netherlands	JQ948575	JQ949896
	CBS 125959	*Fragaria* × *ananassa*, seed	Netherlands	JQ948576	JQ949897
	CBS 126366	*Fragaria* × *ananassa*	USA	JQ948585	JQ949906
	CBS 126367	*Fragaria* × *ananassa*	USA	JQ948586	JQ949907
	CBS 126370	*Fragaria* × *ananassa*	USA	JQ948587	JQ949908
	CBS 126504	*Fragaria* × *ananassa*	South Africa	JQ948595	JQ949916
	CBS 127608	*Fragaria* × *ananassa*	Canada	JQ948594	JQ949915
	CBS 127609	*Fragaria* × *ananassa*	USA	JQ948590	JQ949911
	CBS 127610	*Fragaria* × *ananassa*	USA	JQ948591	JQ949912
	CBS 129933	*Fragaria* × *ananassa*	USA	JQ948592	JQ949913
	CBS 129936	*Fragaria* × *ananassa*	Israel	JQ948582	JQ949903
	CBS 129937	*Fragaria* × *ananassa*	Israel	JQ948583	JQ949904
	IMI 301119	*Fragaria vesca*	Kenya	JQ948596	JQ949917
	IMI 311743	*Fragaria* sp., fruit lesion	USA	JQ948588	JQ949909
	IMI 324995	*Fragaria* × *ananassa*	USA	JQ948589	JQ949910
	IMI 360928	*Fragaria × ananassa*, fruit lesion	Switzerland	JQ948573	JQ949894
	IMI 364856,	*Fragaria × ananassa*, crown	Spain	JQ948574	JQ949895
	IMI 391664	*Fragaria × ananassa*	Israel	JQ948581	JQ949902
	IMI 348177	*Fragaria × ananassa*, crown	USA	JQ948593	JQ949914
	IMI 348497	*Fragaria × ananassa*, crown	France	JQ948570	JQ949891
	CBS 126377	*Fragaria × ananassa*	Netherlands	JQ948563	JQ949884
	IMI 299103	*Fragaria vesca*	UK	JQ948561	JQ949882
	IMI 345032	*Fragaria × ananassa*, fruit	Italy	JQ948571	JQ949892
	IMI 348502	*Fragaria × ananassa*, crown	France	JQ948568	JQ949889
	CBS 127612	*Fragaria* × *ananassa*	USA	JQ948560	JQ949881
	CCTUFN3	*Fragaria × ananassa*, fruit	Iran	KX592833	KX592846
	CCTUFS8	*Fragaria × ananassa*, fruit	Iran	KX592834	KX592847
	CCTUSN1	*Fragaria × ananassa*, stem	Iran	KX592838	KX592852
	CCTUSN2	*Fragaria × ananassa*, stem	Iran	KX592839	KX592853
	CCTUST1	Healthy *Sonchus oleraceus*	Iran	MH250132	MH250122
	CCTUST2	Healthy *Sonchus oleraceus*	Iran	MH250133	MH250123
	CCTUST3	Healthy *Sonchus oleraceus*	Iran	MH250134	MH250124
	CCTUST4	Healthy *Sonchus oleraceus*	Iran	MH250135	MH250125
	CCTUSh1	Health *Fumaria officinalis*	Iran	MH250136	MH250126
	CCTUSh2	Healthy *Fumaria officinalis*	Iran	MH250137	MH250127
	CCTUT	Healthy *Amaranthus viridis*	Iran	MH250138	MH250128
	CCTUK	Healthy *Lactuca serriola*	Iran	MH250139	MH250129
	CCTUP1	Healthy *Convolvulus arvensis*	Iran	MH250140	MH250130
	CCTUP2	Healthy *Convolvulus arvensis*	Iran	MH250141	MH250131
*C. orchidophilum*	CBS 632.80^*^	*Dendrobium* sp.	USA	JQ948481	JQ949802
	CBS 119291	*Cycnoches aureum*	Panama	JQ948484	JQ949805
*C. salicis*	CBS 607.94^*^	*Salix* sp., leaf, spot	Netherlands	JQ948791	JQ950111
	CBS 128556	*Fragaria × ananassa*, fruit rot	New Zealand	JQ948804	JQ950124
	CBS 128557	*Fragaria × ananassa*, fruit rot	New Zealand	JQ948805	JQ950125
	IMI 345581	*Fragaria × ananassa*, lesion	New Zealand	JQ948806	JQ950126
*C. simmondsii*	CBS 122122^*^	*Carica papaya*, fruit	Australia	JQ948606	JQ949927
	CBS 295.67	*Fragaria* sp., fruit	Australia	JQ948608	JQ949929
	IMI 345034	*Fragaria × ananassa*, fruit	Australia	JQ948609	JQ949930

1CBS, ^Culture collection of the Centraalbureau voor Schimmelcultures, Fungal Biodiversity Center, Utrecht, The Netherlands;^ IMI, ^Culture collection of CABI Europe UK Center, Egham, UK;^ CCTU, ^Culture Collection ofTabriz^^University.^

* *Ex^−typeor^ Ex^−epitypestrains^*.

### Pathogenicity Test on Detached Leaves of Strawberry

To fulfill Koch's postulates, a pathogenicity test on detached untreated leaves of *Fragaria* × *ananassa* cv. Elsanta (a susceptible cultivar to strawberry anthracnose). The test was performed to evaluate the pathogenicity of the ten fungal isolates recovered in this study, in comparison to the type strains of *C. nymphaeae, C. acutatum sensu stricto, C. godetiae, C. fioriniae, C. salicis, C. simmondsii* and *C. nymphaeae*. *Colletotrichum nymphaeae* strain Cch32 was used as positive control due to its high aggressiveness on strawberry fruits of *Fragaria* × *ananassa* cv. Elsanta in previous study (Karimi et al., 2017). Detached leaves were first surface-sterilized by dipping in 1 % sodium hypochlorite for 30 s and ethanol 70% followed by rinsing three times in sterile distilled water. The leaves were left to dry on sterile paper under a laminar hood before inoculation. Inoculation was carried out according to Saha et al. (2010), with some modifications. Based on the leaf size, four to 12 wounds were initially made on the adaxial surface of each leaf using a sterile sharp needle. For inoculation, a plug of 5 mm of seven-day-old fungal cultures was placed on the wounds of each leaf. After inoculation, leaves were placed in plastic containers with moistened filter papers at 23°C to maintain high relative humidity (>95%). Five detached leaves were inoculated for each fungal strain and pathogen-free PDA plugs were used as control. After 8 days, the severity of anthracnose disease were recorded and calculated for each strawberry leaf using imageJ v. 1.51i software (https://imagej.nih.gov/ij/index.html).

### Rep-PCR Fingerprinting

A polymorphic analysis based on repetitive-sequence-based PCR (rep-PCR) genomic fingerprinting was carried out for 17 fungal strains in this study using the primers of BOXA1R, REP1R-1/REP2-1, and ERIC1R/ERIC2 (Versalovic et al., 1991, 1994). PCR reactions were repeated twice (independent reactions) to confirm reproducibility of the results. PCR reaction mixtures were prepared in 25 ml volumes as follows: 12.5 μl 1X Dream Taq Green PCR Mastermix (Fer-mentas) 9.5 μl sterile distilled water, 1 μl each of each primer (0.2 μmol/μl stock) and 1 μl of template DNA (50 ng). DNA-free water (nuclease free) was used as a control. The PCR cycles were carried out as follows: an initial denaturation at 96 °C for 5 min, followed by 30 cycles at 94°C for 60 s, 40°C (REP primers) or 51°C (ERIC/BOX primers) for 1 min and 65°C for 8 min and a final extension at 65°C for 8 min. An aliquot of 5 μl PCR product of each isolate was loaded onto 1.5 % agarose gel 1 × Tris-Acetate-EDTA (TAE) buffer at 100V for 2.5 h and then stained with ethidium bromide. Amplicons size was determined in comparison to the GeneRuler^TM^ 1kb DNA ladder. Unambiguous bands were scored as “0” and “1” to make a matrix data according to their presence or absence. UPGMA analysis was carried out for each marker using a simple matching coefficient with NTSYS-PC v2.2 and finally a consensus dendrogram was built.

### Statistical Analyses

One-way ANOVA was performed to analyze results of pathogenicity test using SAS software package (SAS® software v. 9.1.3 SP4 portable). Least significant difference (LSD) was used to compare the means.

## Results

### Isolation

Of the various weeds sampled in the strawberry fields, 10 fungal isolates with a colony morphology resembling the one of *Colletotrichum* were recovered. These isolates were obtained from the leaves of asymptomatic plants of *Amaranthus viridis* L. (one isolate), *Convolvulus arvensis* L. (two isolates), *Fumaria officinalis* L. (two isolates), *Lactuca serriola* L. (one isolate) and *Sonchus oleraceus* L. (four isolates).

### Morphological Description

**Figure 1:**
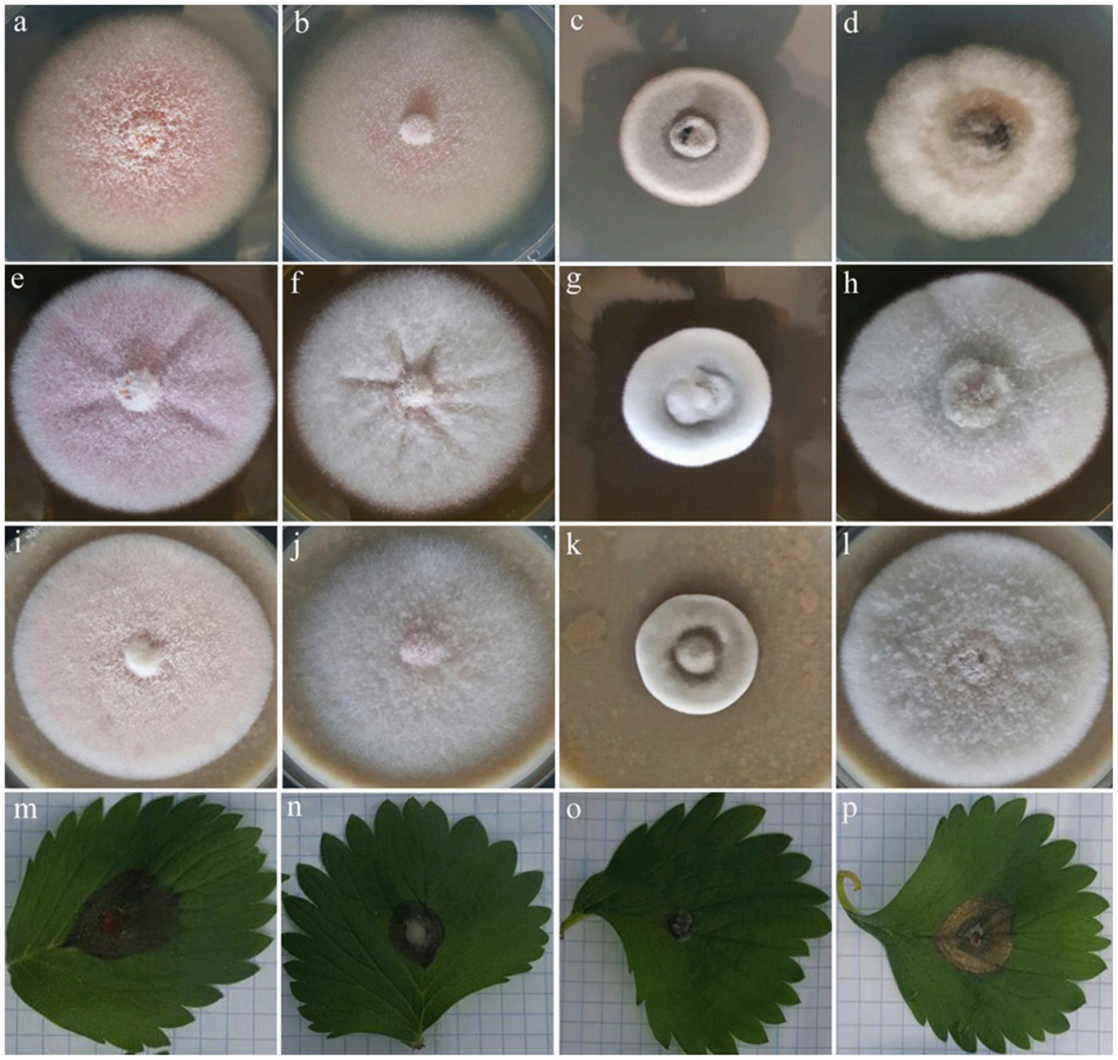
Colony morphology of four fungal strains of *C. acutatum* (CBS 112996), *C. salicis* (CBS 607.94), *C. simmondsii* (CBS 122122) and *C. fioriniae* (CBS 128517) on PDA **(a,b,c,d)**, MEA **(e,f,g,h)**, and OA **(i,j,k,l)**, respectively plus their pathogenicity on strawberry leaves of *Fragaria* × *ananassa* cv. Elsanta **(m,n,o,p)** respectively.

**Figure 2:**
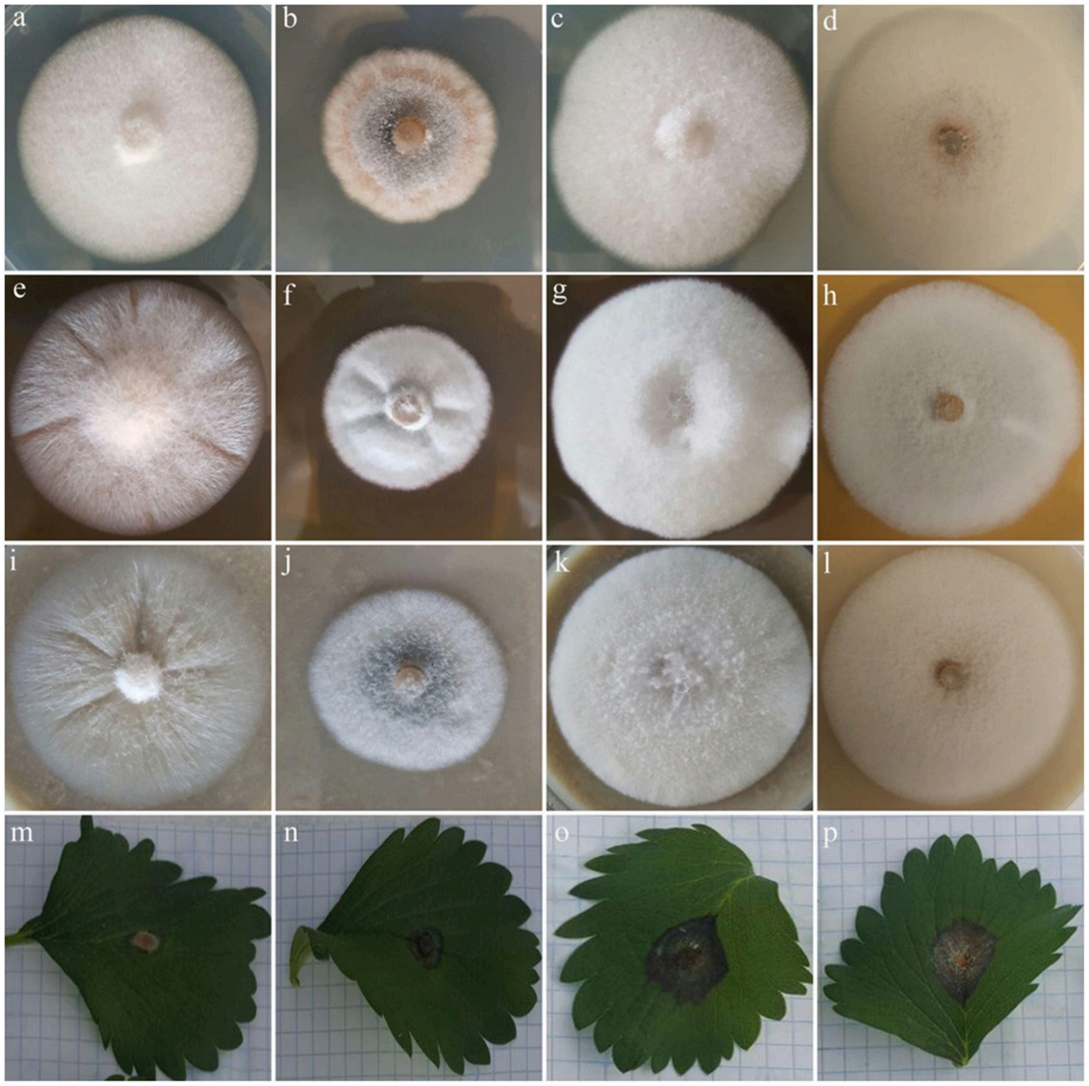
Colony morphology of four fungal strains of *C. godetiae* (CBS 133.44), *C. nymphaeae* (CBS 515.78), *C. nymphaeae* CCTUCch32 and *C. nymphaeae* CCTUK on PDA **(a,b,c,d)**, MEA **(e,f,g,h)**, and OA **(i,j,k,l)**, respectively plus their pathogenicity on strawberry leaves of *Fragaria* × *ananassa* cv. Elsanta **(m,n,o,p)**, respectively.

Macro morphological features of type strains and isolates recovered from common weeds of strawberry fields are represented in Table 2. All isolates obtained from weeds were morphologically similar to each other and to *C. nymphaeae* strain CCTUCch32 reported from strawberry (Table 2, **Figures 2c,d,g,h,k,l**). On the contrary, colony morphology and growth rate of these strains were almost different compared to the type strain of *C. nymphaeae* (CBS 515.78) (Table 2, **Figures 2b–d,f–h,j–l**). Other fungal strains of *Colletotrichum* spp. in this study shared some similar characteristics (colony morphology and growth rate) compared to isolates recovered from weeds in this study (Table 2, Figures 1, 2).

**Table 2:** Colony morphology and morphometry of fungal strains recovered from weeds and type strains belonging to *C. acutatum* complex.

**Fungal strains**	**PDA**		**MEA**		**OA**	
	**CD (mm)**	**CM**	**CD (mm)**	**CM**	**CD (mm)**	**CM**
*C. acutatum* (CBS 112996)^*^	55	R, C, Wa, P	54	R, C, Wa, P, S	59	R, C, Wa, P, S
*C. fioriniae* (CBS 128517)^*^	45	IR, L, Wa, W (m) to B (c)	59	R, C, Wa, W to G	65	R, C, Wa, W to G
*C. godetiae* (CBS 133.44)^*^	55	R, C, Wa, W to P	51	R, C, Wa, W to P, S	59	R, C, Wa, S
*C. nymphaeae* (CBS 515.78)^*^	31	R, C, Wa, G (c) to W (m)	30	R, C, Wa, G, S	35	R, C, Wa, G, S
*C. nymphaeae* CCTUCch32^**^	53	R, C, Wa, W to P	50	R, C, Wa, W to P	64	R, C, Wa, W to G
*C. nymphaeae* CCTUST1	54	R, C, Wa, W to G	53	R, C, Wa, W to G	63	R, C, Wa, W to G
*C. nymphaeae* CCTUST2	55	R, C, Wa, W to G	53	R, C, Wa, W to G	60	R, C, Wa, W to G
*C. nymphaeae* CCTUST3	54	R, C, Wa, W to G	52	R, C, Wa, W to G	62	R, C, Wa, W to G
*C. nymphaeae* CCTUST4	54	R, C, Wa, W to G	53	R, C, Wa, W to G	61	R, C, Wa, W to G
*C. nymphaeae* CCTUSh1	56	R, C, Wa, W to G	53	R, C, Wa, W to G	63	R, C, Wa, W to G
*C. nymphaeae* CCTUSh2	59	R, C, Wa, W to G	53	R, C, Wa, W to G	63	R, C, Wa, W to G
*C. nymphaeae* CCTUK	56	R, C, Wa, W to G	53	R, C, Wa, W to G	62	R, C, Wa, W to G
*C. nymphaeae* CCTUT	50	R, C, Wa, W to G	55	R, C, Wa, W to G	62	R, C, Wa, W to G
*C. nymphaeae* CCTUP1	52	R, C, Wa, W to G	52	R, C, Wa, W to G	60	R, C, Wa, W to G
*C. nymphaeae* CCTUP2	55	R, C, Wa, W to G	53	R, C, Wa, W to G	60	R, C, Wa, W to G
*C. salicis* (CBS 607.94)^*^	62	R, C, Wa, P	62	R, C, Wa, P	59	R, C, Wa, W to G
*C. simmondsii* (CBS 122122)^*^	28	R, C, Wa, G (c) to W (m)	26	R, C, Wa, G (c) to W (m)	26	R, C, Wa, G (c) to W (m)

* Ex-type and ex-epitype strain,

** *fungal strain obtained from strawberry. (c), center; C, circular; CD, colony diameter (average), CM, colony morphology; G, gray; L, lobate; (m), margin; P, pink; IR, irregular; R, regular, S, sector; W, white, Wa, without aerial mycelium*.

### Phylogenetic Analysis

**Figure 3:**
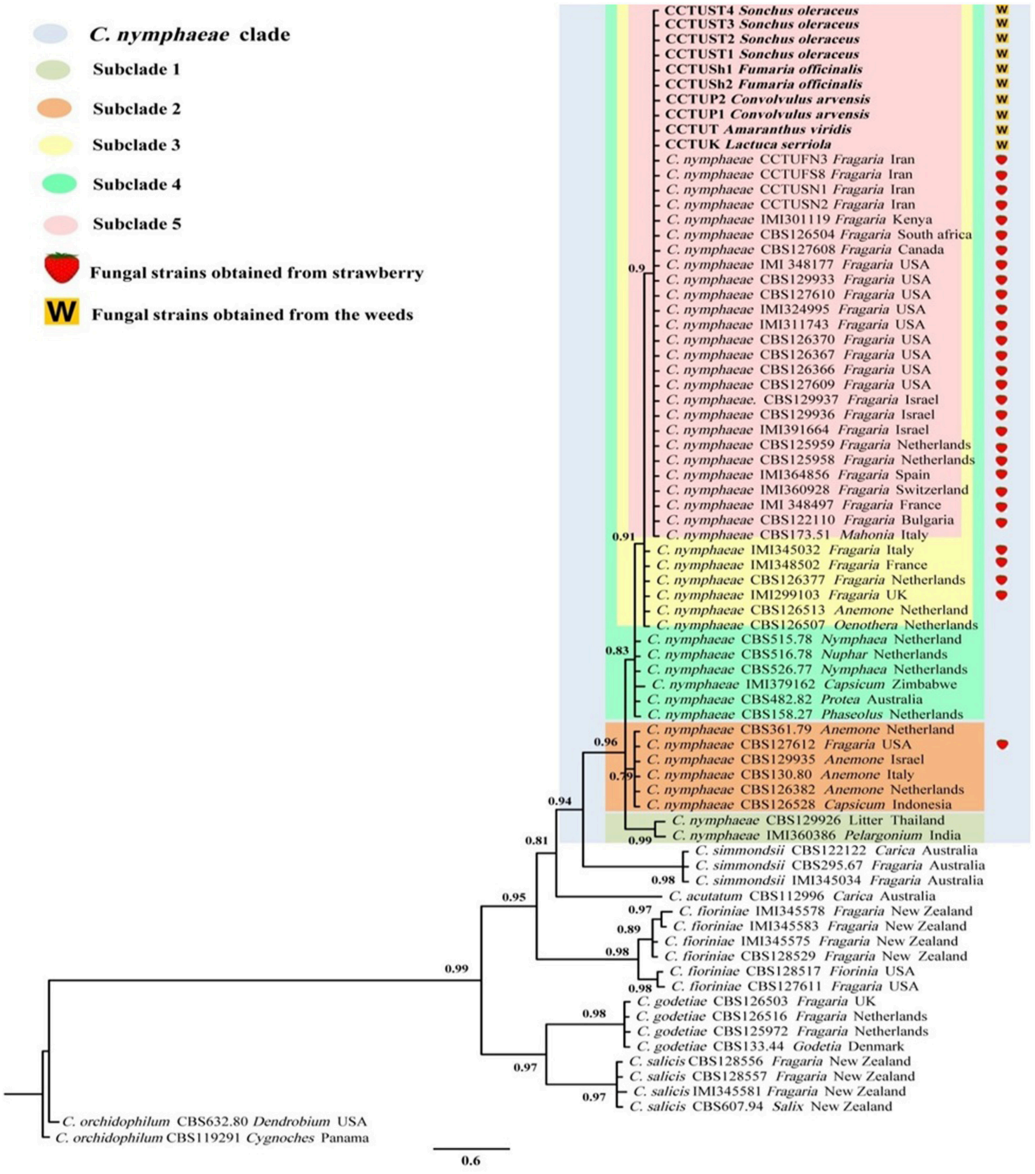
Bayesian inference phylogenetic tree of the *Colletotrichum* spp. reported from strawberry belonging to *C. acutatum* species complex. The tree was built using concatenated sequences of GAPDH (*glyceraldehyde 3*-*phosphate dehydrogenase*) and TUB2 (*beta-tubulin*), with the GTR+G+I model. Two strains of *C. orchidophilum* (CBS 632.80, CBS 119291) were used as out-group. The scale bar shows 0.6 expected changes per site. Strawberry isolates are labeled with a strawberry and the isolates recovered from the weeds in this study are marked with a W.

The final aligned TUB and GADPH datasets contained 74 in-group taxa with a total of 757 (gene boundaries, GAPDH: 1–265, TUB: 266–757) characteristics containing 109 and 94 unique site patterns, respectively. MrModeltest v. 2.3 found GTR+G+I to be the most fitting replacement models for both concatenated datasets. Bayesian inference of both concatenated datasets placed all the isolates sequenced in this study in the *C. nymphaeae* clade with the highest posterior probability (Figure 3). The *C. nymphaeae* clade with high posterior probability contained five subclades. All the isolates generated in this study were placed in subclade S5 containing the strains reported from strawberry plants besides one isolate from *Mahonia* sp. in Italy.

### Pathogenicity Test

**Figure 4:**
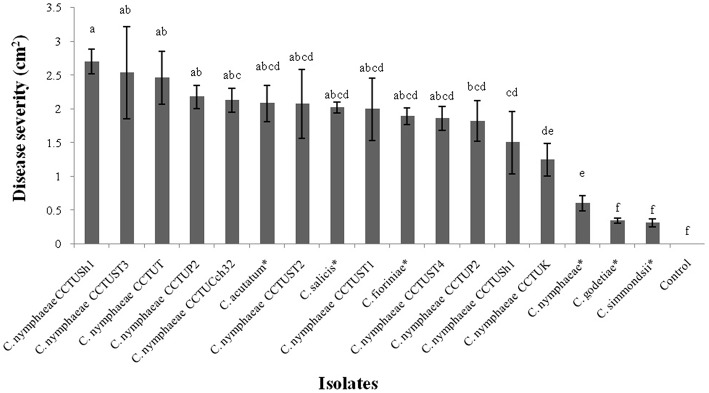
Disease severity of anthracnose on strawberry leaves of *Fragaria* × *ananassa* cv. Elsanta caused by the *Colletotrichum* spp. in this study. The mean with the common letters have no significant differences at level of *p* < 0.01. Error bars show standard error of the means. ^*^Ex-type or ex-epitype strains.

Pathogenicity test revealed that all isolates obtained from weeds in this study and other type strains of *Colletotrichum* spp. were able to induce anthracnose symptoms on strawberry leaves of *Fragaria* × *ananassa* cv. Elsanta (Figures 1, 2). However, there was a significant difference between fungal strains in terms of disease severity (df = 17, *F* = 6.95, *p* = < 0.0001). *Colletotrichum nymphaeae* CCTUSh1 showed the highest pathogenicity, although it had no significant difference with most strains such as the type strains of *C. acutatum, C. salicis* and *C. fioriniae* (Figures 1, 2, 4). The type strain of *C. nymphaeae* had significantly the lowest virulence compared to other *C. nymphaeae* strains in this study besides *C. nymphaeae* CCTUK (Figure 4). Moreover, *C. nymphaeae* strain CCTUCch32 after four strains of CCTUSh1, CCTUST3, CCTUT, and CCTUP1 had the highest pathogenicity compared to others (Figure 4). Type strains of *C. godetiae* and *C. simmondsii* showed the lowest pathogenicity and had no significant difference with healthy control (Figure 4).

### Repetitive-Sequence-Based PCR

**Figure 5:**
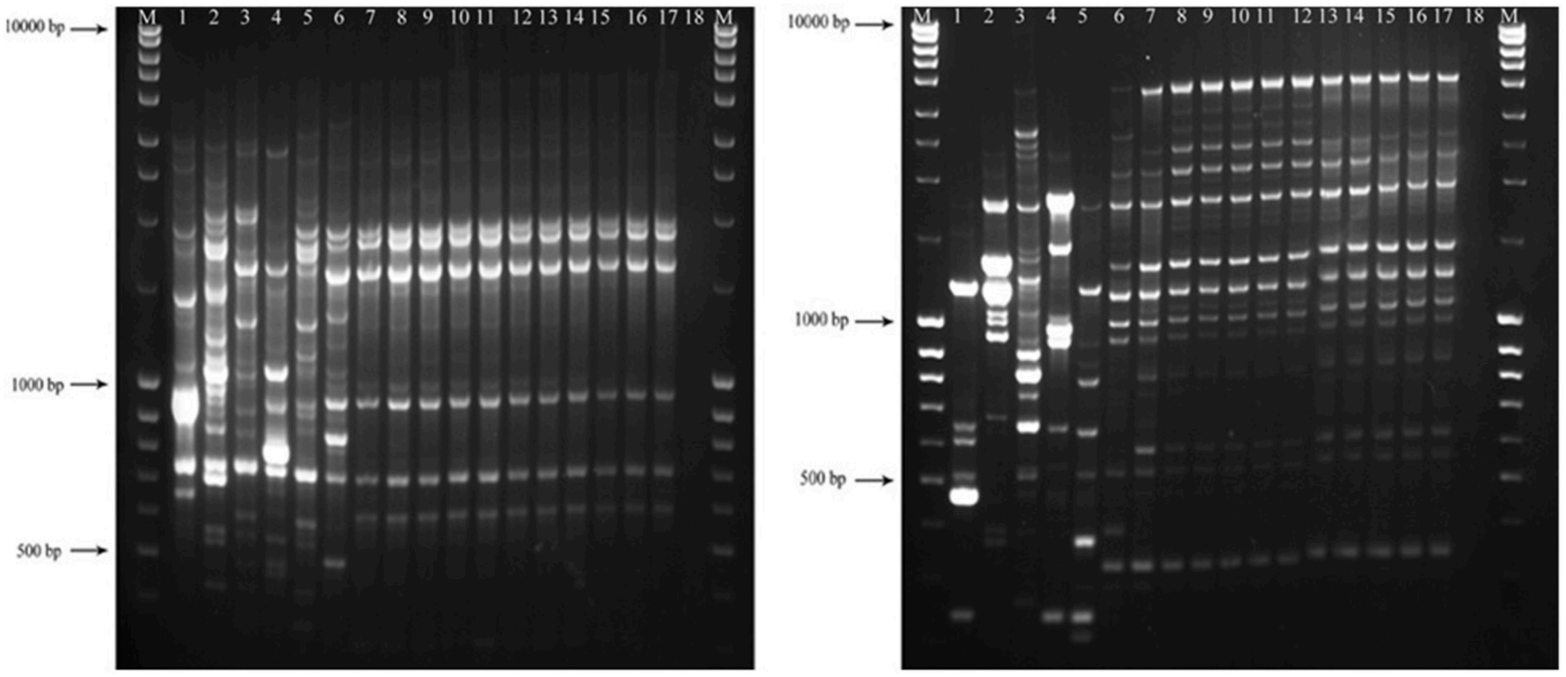
Amplified profiles of genomic DNA belong to *Colletotrichum* spp. generated with BOX (left-hand image) and ERIC1R/ERIC2 (right-hand image) primers. The first and the last columns (M) show GeneRuler Ladder bands (#SM0403). The lanes with consecutive numbers from one to seventeen correspond to *C. acutatum* (CBS 112996), *C. salicis* (CBS 607.94), *C. fioriniae* (CBS 128517), *C. godetiae* (CBS 133.44), *C. simmondsii* (CBS 122122), *C. nymphaeae* (CBS 515.78), *C. nymphaeae* CCTUCch32, *C. nymphaeae* CCTUST1, *C. nymphaeae* CCTUST2, *C. nymphaeae* CCTUST3, *C. nymphaeae* CCTUST4, *C. nymphaeae* CCTUK, *C. nymphaeae* CCTUT, *C. nymphaeae* P1*, C. nymphaeae* CCTUP2, *C. nymphaeae* CCTUSh1 and *C. nymphaeae* CCTUSh2. The eighteenth well represents negative control.

**Figure 6:**
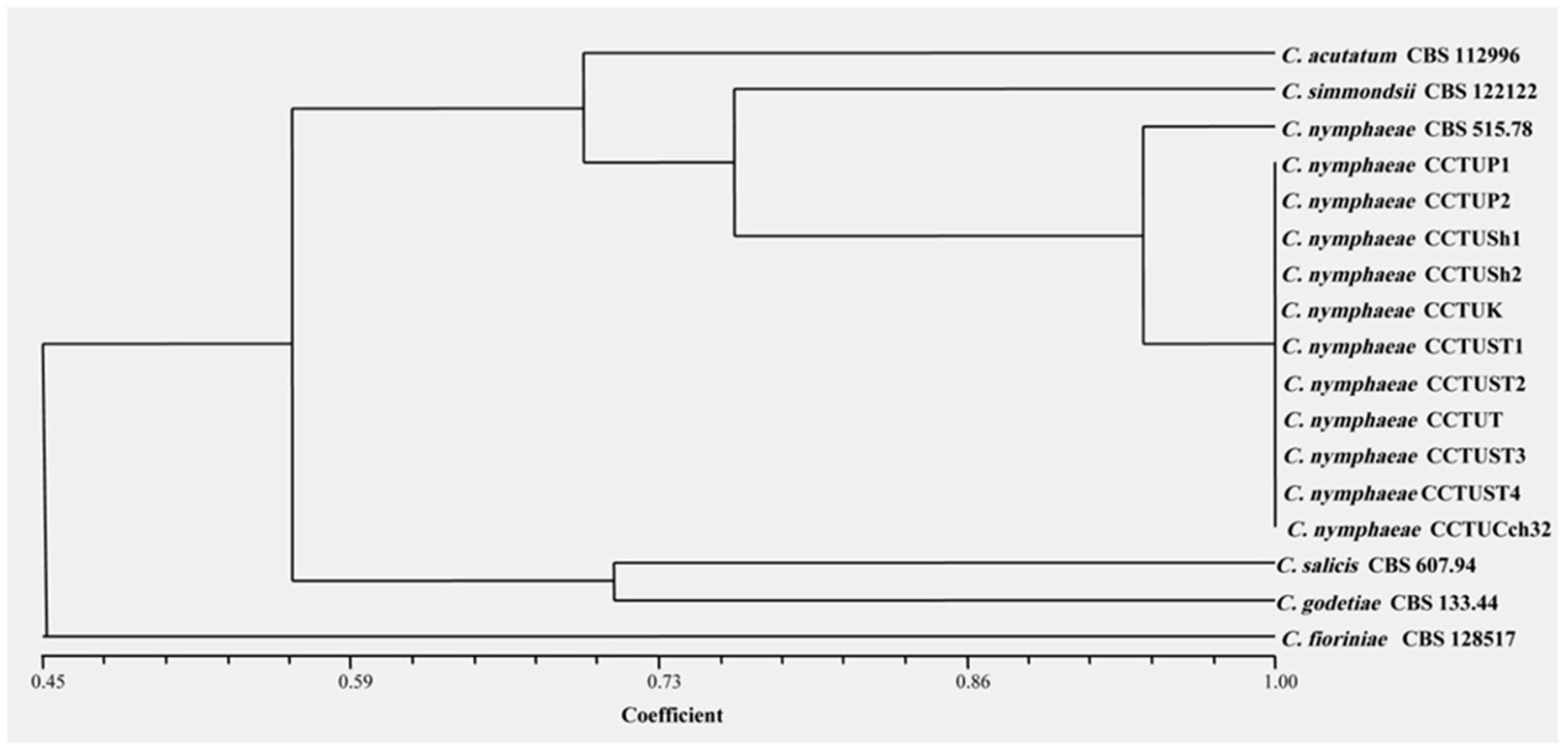
Dendrogram generated with DNA banding patterns of BOX primer using UPGMA cluster analysis. Similarity between the patterns was calculated with simple matching coefficient. Ex-type and Ex-epitype cultures are represented with CBS mark.

**Figure 7:**
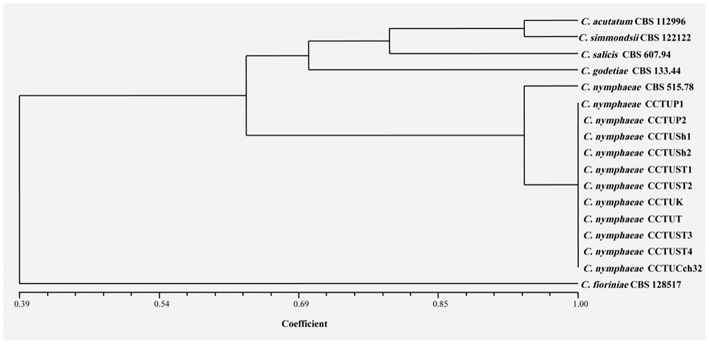
Dendrogram generated with DNA banding patterns of ERIC1R/ERIC2 primer using UPGMA cluster analysis. Similarity between the patterns was calculated with simple matching coefficient. Ex-type and Ex-epitype cultures are represented with CBS mark.

**Figure 8:**
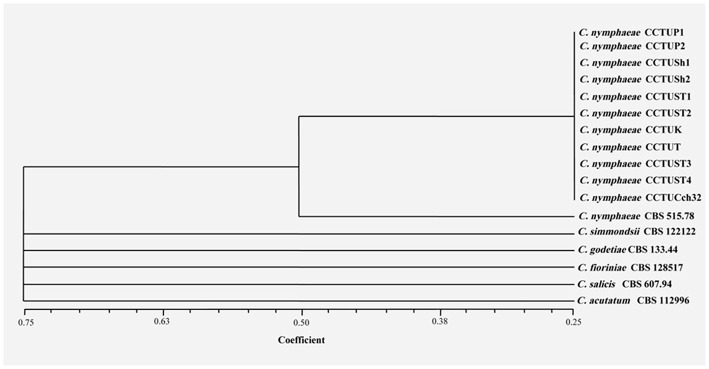
Consensus dendrogram generated with combined DNA banding patterns of BOX and ERIC1R/ERIC2 primers using UPGMA cluster analysis. Similarity between the patterns was calculated with simple matching coefficient. Ex-type and Ex-epitype cultures are represented with CBS mark.

No amplification was observed for primer set REP1R-1/REP2-1 based on repetitive-sequence-based PCR analysis after several attempts while two primer sets, BOXA1R and ERIC1R/ERIC2, amplified the genomic DNA with reproducible DNA fingerprints (Figure 5). The size of amplified amplicons for each primer set ranged from 400 to 5,000 bp and from 200 to 6,000 bp, respectively (Figure 5). UPGMA clustering analysis of individual and combined data matrix generated with the amplicon patterns of BOXA1R and ERIC1R/ERIC2 placed all strains of this study at level of species showing the efficacy of these primers to generate species-specific DNA fingerprints even in the case of cryptic species (Figures 6–8). Individual and consensus dendrograms resided the type strain of *C. nymphaeae* as subclade compared to other *C. nymphaeae* strains obtained from weed plants and strawberry (Figures 6–8).

## Discussion

This study confirmed the hypothesis that weeds present in strawberry fields can host *C. nymphaeae* as a potential inoculum reservoir. The characterization of the *Colletotrichum* isolates recovered from the common weeds across the strawberry fields in Iran, indicates that weed isolates shared similar macro morphological characteristics (Table 2, Figure 2) and were identical to *C. nymphaeae* CCTUCch32 isolated from symptomatic strawberry (Karimi et al., 2017). On the other hand, in terms of colony features and growth rate, the 10 isolates showed some discrepancies with type strain of *C. nymphaeae* from *Nymphaea alba* (Table 2, Figures 1, 2). Moreover, they shared some features with other type strains including *C. acutatum, C. salicis, C. simmondsii, C. fioriniae* and *C. godetiae* (Table 2, Figures 1, 2), indicating the variability and unreliability of macro-morphological characterization for species delimitation. However, Bayesian inference of concatenated alignments of TUB and GAPDH clustered our isolates in *C. nymphaeae* clade inside subclade 5, containing other *C. nymphaeae* strains reported from strawberry worldwide including Iran such as *C. nymphaeae* FN3, FS8, SN1, and SN2 (Karimi et al., 2017). Both TUB and GAPDH genes have been recommended as appropriate barcode genes to reveal cryptic species of *C. acutatum* complex (Damm et al., 2012a). Base on that, using a combination of molecular and morphological characterizations, the 10 *Colletotrichum* sp. isolates from symptomless plants of *A. viridis, C. arvensis, F. officinalis, L serriola*, and *S. oleraceus* were identified as *C. nymphaeae* (Figures 2, 3). This result was also in line with multi-gene and fingerprinting analyses where type strains of *C. nymphaeae* resided in a different subclade (Figures 3, 6–8). Furthermore, in the pathogenicity test, type strain of *C. nymphaeae* showed the lowest virulence on strawberry leaves compared to other *C. nymphaea* strains, except for *C. nymphaeae* CCTUK (Figure 3). Overall, these results further confirm our previous findings (Karimi et al., 2017) that subclade 5 containing strains recovered from strawberry are evolutionary different and constitute a distinct subpopulation of *C. nymphaeae* with host-preference on strawberry. Moreover, this variability in the aggressiveness of a species within *C. acutatum* complex could be associated with the capability of different populations of these species to infect a wider range of hosts and the susceptibility to various environmental conditions (Baroncelli et al., 2015).

Molecular typing based on rep-PCR revealed that this technique can generate species-specific DNA fingerprints, at least for *Colletotrichum* spp. causing strawberry anthracnose belonging to *C. acutatum* complex (Figures 5–8). It is worth noting that dendrograms resulted from BOX, REP1R-1/REP2-1 and their combination showed high similarity with multi-gene tree generated with both TUB and GAPDH genes particularly one resulted from BOX (Figures 3, 6–8). Accordingly, amplified patterns generated by rep-PCR fingerprinting analysis revealed that all isolates recovered from weeds were highly similar to *C. nymphaeae* CCTUCch32 isolated from strawberry, while they showed some discrepancies with the type strain of *C. nymphaeae* (Figure 5). Similarly to multi-gene analysis, the type strain of *C. nymphaeae* resides in a distinct subclade (Figures 6–8). Based on the dendrograms of this study, rep-PCR confirmed to be an appropriate molecular marker to reveal intra and inter species variation of *Colletotrichum* spp. causing anthracnose on strawberry. It seems that this molecular technique can reveal all cryptic species among *C. acutatum sensu lato*, however to further confirm this hypothesis, more type and epi-type fungal strains belong to *C. acutatum sensu lato* should be investigated. Although rep-PCR fingerprinting analysis has been originally used for molecular typing and identification of bacterial species (Versalovic et al., 1991, 1994), the efficacy of this molecular typing technique in unveiling of fungal cryptic species belonging to various fungal complex species was shown in some studies (Alves et al., 2007; Faedda et al., 2011). However, this is the first time that rep-PCR is used as authentic molecular typing technique for unveiling *Colletorichum* spp. causing strawberry anthracnose belonging to the *C. acutatum sensu lato*. This result is consistent with a previous study (Faedda et al., 2011) where dendrograms obtained with RAPD-PCR analysis, a molecular technique very similar to Rep-PCR (Gillings and Holley, 1997), were showed to be almost identical, and even more informative of intraspecific variability, compared to ITS- and TUB-based UPGMA phylogetic trees of isolates of the *C. acutatum* species complex from various host plants, including strawberry.

To the best of our knowledge, this is the first time that asymptomatic plants of *A. viridis, C. arvensis, F. officinalis, L*. *serriola*, and *S. oleraceus* are reported as possible inoculum reservoirs of *C. nymphaeae*. In this study, fungal isolates were isolated using the protocol which is commonly used for the isolation of endophytic fungi, highlighting their either endophytic or long lasting biotrophic nature inside those plants. A broad diversity of approaches including biotrophs, nectrotrophs, hemibiotrophs to endophytes are employed by *Colletotrichum* species to colonize and obtain nutrients from their hosts (Baroncelli et al., 2017). Previously, only asymptomatic plants of the weed genera *Vicia* and *Conyza* were reported as inoculum reservoirs of *C. acutatum sensu lato* (Freeman et al., 2001) and the pathogenicity of *C. acutatum sensu lato* was demonstrated on some other weeds in strawberry fields in UK, including *Malva sylvestris, Ranunchulus repens, Sinapis arvensis, Rumex obtusifolius, Geranium dissectum, Polygonatum aviculare*, and *Plantago lanceolata* (Berrie and Burgess, 2003). It is noteworthy that the aggressiveness of four *C. nymphaeae* strains recovered from weeds including *C. nymphaeae* CCTUSh1, CCTUST3, CCTUT, and CCTUP2 was higher than *C. nymphaeae* CCTUCch32, which was the most aggressive strain in our previous studies (Karimi et al., 2017). These findings highlight that the infected weeds close to strawberry fields can possibly act as a potential reservoirs of pathogen inoculum during crop season, as highlighted by Berrie and Burgess (2003). In view of the fact that no specific resting structure, which can let it to survive in buried plant residues, has so far been reported for *C. acutatum sensu lato* (Ureña-Padilla et al., 2001; Freeman, 2008), weed plants identified in this study could allow/contribute to the survival of the pathogen between seasons. Therefore, the removal of weeds that could act as potential pathogen reservoirs should be considered to optimize disease management.

## Conclusion

The presence of this pathogen on common weeds growing close to or within strawberry fields indicates that they may serve as a reservoir of inoculum and calls for further practical investigations to assess the advantage of their removal to reduce pathogen inoculum and improve the efficacy of anthracnose disease control on strawberry. Moreover, our study demonstrates that, when the type strains of each species are available, rep-PCR is able to reveal intra and inter species variation of fungal species belonging to the *C. acutatum* complex, even in case of cryptic species.

## Author Contributions

KK designed and performed the experiments, analyzed and discussed the results, and wrote the manuscript. MA and IP discussed the results and wrote the manuscript.

### Conflict of Interest Statement

The authors declare that the research was conducted in the absence of any commercial or financial relationships that could be construed as a potential conflict of interest.
