# Supplementary figures and images for: Weeds as Potential Inoculum Reservoir for Colletotrichum nymphaeae Causing Strawberry Anthracnose in Iran and Rep-PCR Fingerprinting as Useful Marker to Differentiate C. acutatum Complex on Strawberry
Source: Front Microbiol. 2019 Feb 12;10:129. doi: 10.3389/fmicb.2019.00129 (PMC6379352; doi:10.3389/fmicb.2019.00129)

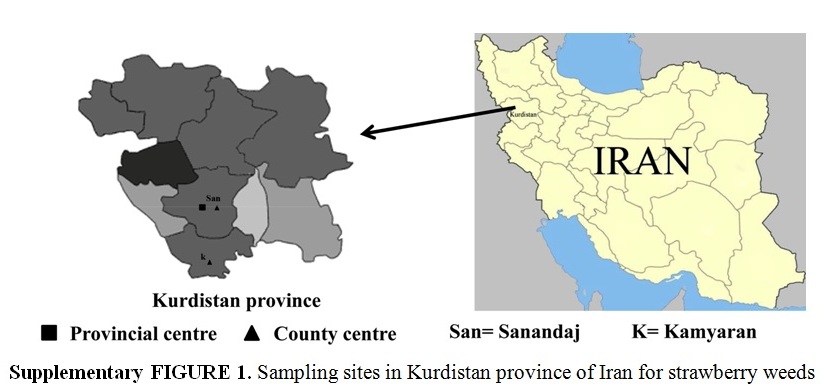

Supplement: Supplementary file 1 [file Image_1.jpg]
